# Recurrent calf myositis as revealing manifestation of Crohn disease: a case report

**DOI:** 10.3389/fped.2025.1676609

**Published:** 2025-12-18

**Authors:** Anna La Rosa, Alessandra Meneghel, Chiara Giraudo, Pietro Zucchetta, Francesca Tirelli, Francesca Trevisan, Fiorella Calabrese, Francesco Zulian

**Affiliations:** 1 Department of Women’s and Children’s Health, University of Padova, Padua, Italy; 2 Unit of Advanced Clinical and Translational Imaging, Department of Cardiac, Thoracic, Vascular Sciences and Public Health– DCTV, University of Padova, Padua, Italy; 3 Unit of Nuclear Medicine, Department of Medicine – DIMED, University of Padova, Padoua, Italy; 4 Department of Cardiac, Thoracic, Vascular Sciences and Public Health, University of Padova, Padova, Italy

**Keywords:** calf myositis, crohn's disease, extraintestinal manifestation IBD, myositis, NOD2, SARS- CoV-2

## Abstract

Crohn's disease (CD) is a chronic inflammatory bowel disease (IBD) that may present in children with extraintestinal manifestations (EIM). Inflammatory myopathies are rarely described in this context. Herein we describe the case of a teenager with recurrent episodes of calf myositis, also known as “Gastrocnemius Myalgia Syndrome” (GMS), as the only manifestation of CD in a pre-clinical phase. A 15-year-old girl presented three episodes of acute bilateral calf myositis with inability to walk over a period of three years. Each episode was preceded by febrile upper respiratory tract infections, with levels of IgG for Sars-Cov2 compatible with a recent infection. Clinical and laboratory tests showed an acute inflammatory state which, in the first two episodes, spontaneously improved in about 20 days. During the third episode, a whole-body PET-MRI detected hypermetabolism, associated with muscle edema of the posterior compartment of the legs and thickening of the terminal ileum with significant tracer uptake. Intestinal and muscle biopsies confirmed the diagnosis of CD and focal myositis. The patient was treated with corticosteroids and azathioprine with rapid resolution of pain, fever and intestinal inflammation. Our case clearly shows that GMS should be considered among the EIM of pediatric CD and can precede IBD onset by years. Therefore, CD should be ruled out in all pediatric patients presenting with predominant, recurrent calf myositis.

## Introduction

Up to 50% of patients with inflammatory bowel disease (IBD) experience at least one extraintestinal manifestation (EIM) during the course of their illness, presenting either synchronously or metachronously with respect to IBD. EIMs can affect any organ system and may be a source of considerable morbidity and, in some cases, even mortality (1). Among the most common are immune-mediated arthropathies, along with mucocutaneous, ocular, and hepatobiliary disorders (2). Inflammatory myopathies (IM) have been rarely reported in association with CD and mainly consist of orbital myositis or dermatomyositis (3, 4).

Herein we describe the case of a teenager with a three-year history of recurrent isolated episodes of myositis selectively affecting calf muscles, known in adults as “gastrocnemius myalgia syndrome” (GMS) (5), as sole presentation of CD, and provide a review of the literature.

## Case description

A 15-year-old female presented, over a three-year timeframe, three episodes of fever and acute severe bilateral calf pain, resulting in inability to walk. In all episodes, the onset of calf pain was preceded by febrile upper respiratory tract infections (URTI).

At the first episode, the patient, admitted to a peripheral hospital, had high-degree fever and acute bilateral calf pain, initially thought to be associated with a recent surgery for clubfoot correction. She also reported a contact with a COVID-19-positive individual four weeks prior to the onset of symptoms. Physical examination was unremarkable except for very painful calves on palpation and movement, with impaired ambulation. Overall muscle strength was preserved.

**Figure 1:**
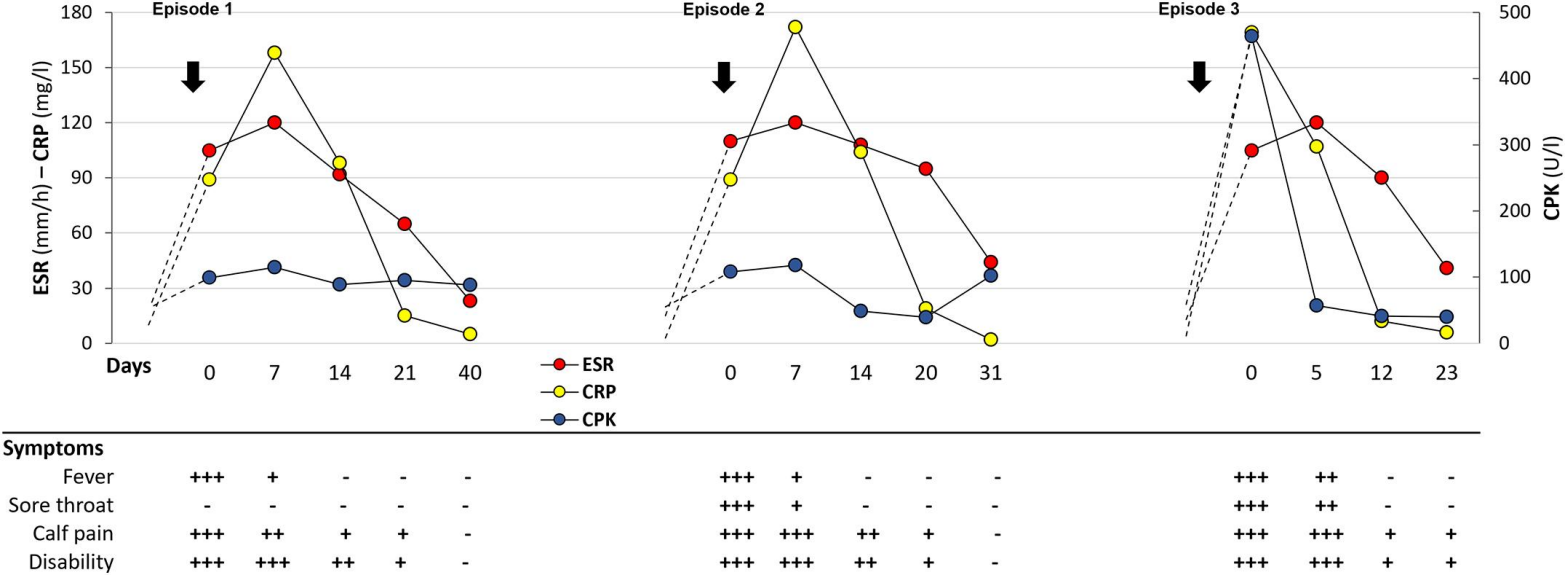
Timeline of the clinical features and laboratory changes during the three episodes of gastrocnemius myalgia syndrome.

Blood tests revealed an inflammatory profile (WBC count 15.9 × 10^3^/μL, PMNc: 78%, Hgb: 11.2 g/L, PLT: 517,000, ESR: 120 mm/h, CRP: 158 mg/L), while CPK, ALT and AST were within normal values for age. Searches for viral infections, including EBV, CMV, Adeno and Parvovirus B19, were all negative. A lower limb ultrasound showed a fascial fluid collection on the right medial gastrocnemius. Broad-spectrum antibiotics were started with resolution of myalgias and normalization of laboratory abnormalities in about 20 days (Figure 1). The patient was then referred to our Pediatric Rheumatology Academic Center for a second opinion after resolution of this episode of “atypical” myositis. Since then, she has been monitored at our Center approximately every 4–6 months and his parents were encouraged to report any clinical changes.

**Figure 2:**
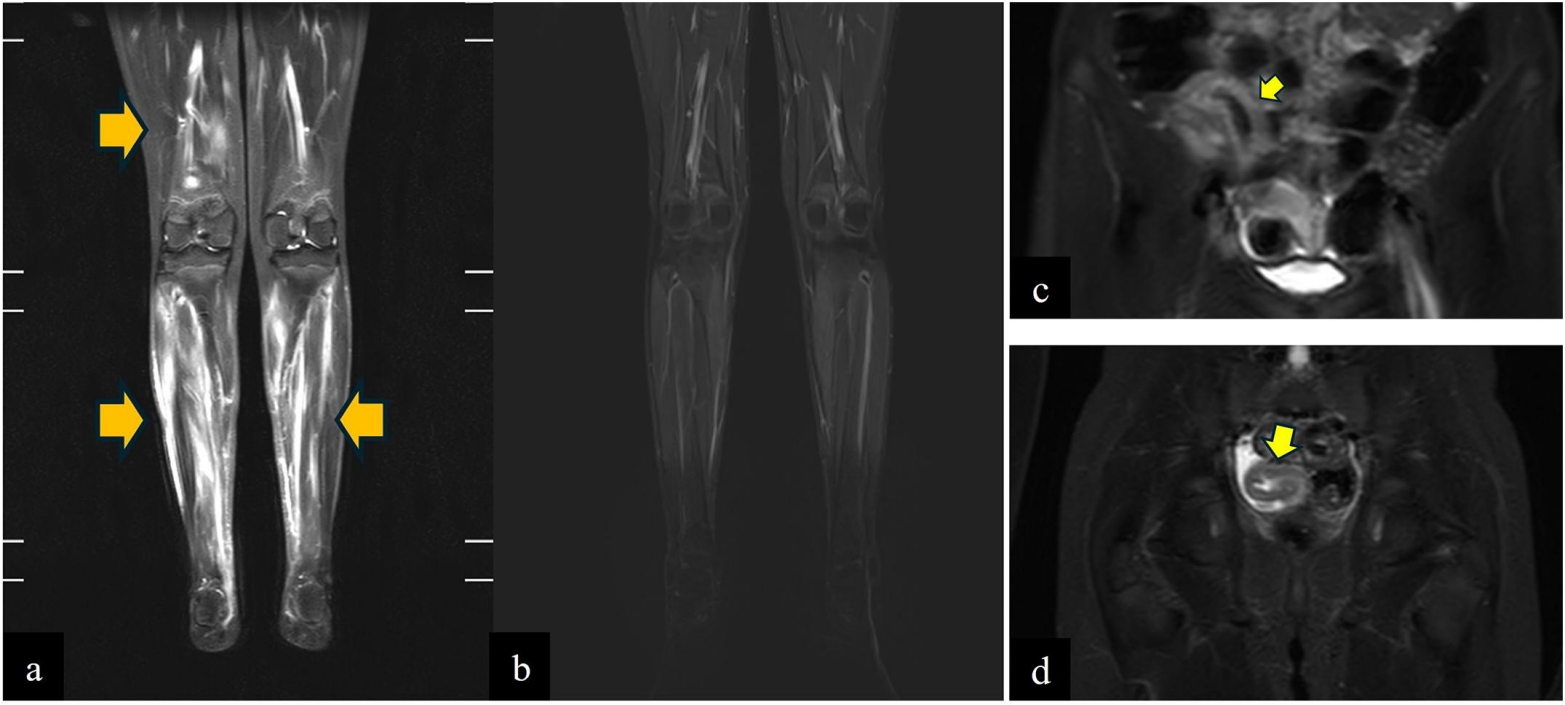
Lower limb coronal STIR showing **(a)** severe muscle edema in the calves and in the muscles of the distal part of the right thigh (orange arrows), **(b)** the resolution of the muscle edema; **(c,d)** thickening and edema of the terminal ileal loop walls (yellow arrows).

Eighteen months later, the patient again presented high-grade fever, intense calf pain, sore throat, and transient diarrhoea. Bacterial cultures from stool and throat swab were negative while viral serology was consistent with a recent Sars-CoV2 infection (6) (serum IgG: 153.5 kBAU/L). Inflammatory markers were markedly elevated (WBC count 22.7 × 10^3^/μL, PMNc: 76%, haemoglobin 10.4 g/L, ESR: 120 mm/h, CRP: 184 mg/L) albeit with normal muscle enzyme levels (CPK: 108 U/L). A significant increase of serum TNF, IL-6 and IL-1α cytokines was also detected. Myositis-specific autoantibody profile was negative while a genetic test for autoinflammatory syndromes showed a heterozygous variants in the *NLRP12 ((NM_144687): c.1206C>G (p.F402L))* and NOD2 genes ((NM_022162): c.3019dup (p.L1007Pfs*2)). A whole-body Magnetic Resonance Imaging (WBMRI) showed severe bilateral and symmetrical edema of the calf muscles and mild edema of the distal muscles of the right thigh, compatible with myositis (Figure 2a), and mild thickening of the last ileal loop (Figures 2c,d). During hospitalization, febrile peaks progressively decreased and resolved spontaneously within 20 days, without therapy. This was accompanied by reduction in myalgia, normalization of inflammatory markers and gradual recovery of normal gait (Figure 1). One month later, a follow-up whole-body MRI showed a complete resolution of the myositis (Figure 2b) and a reduction of the intestinal inflammation. Given the complete clinical and laboratory remission, no further investigations were deemed necessary.

**Figure 3:**
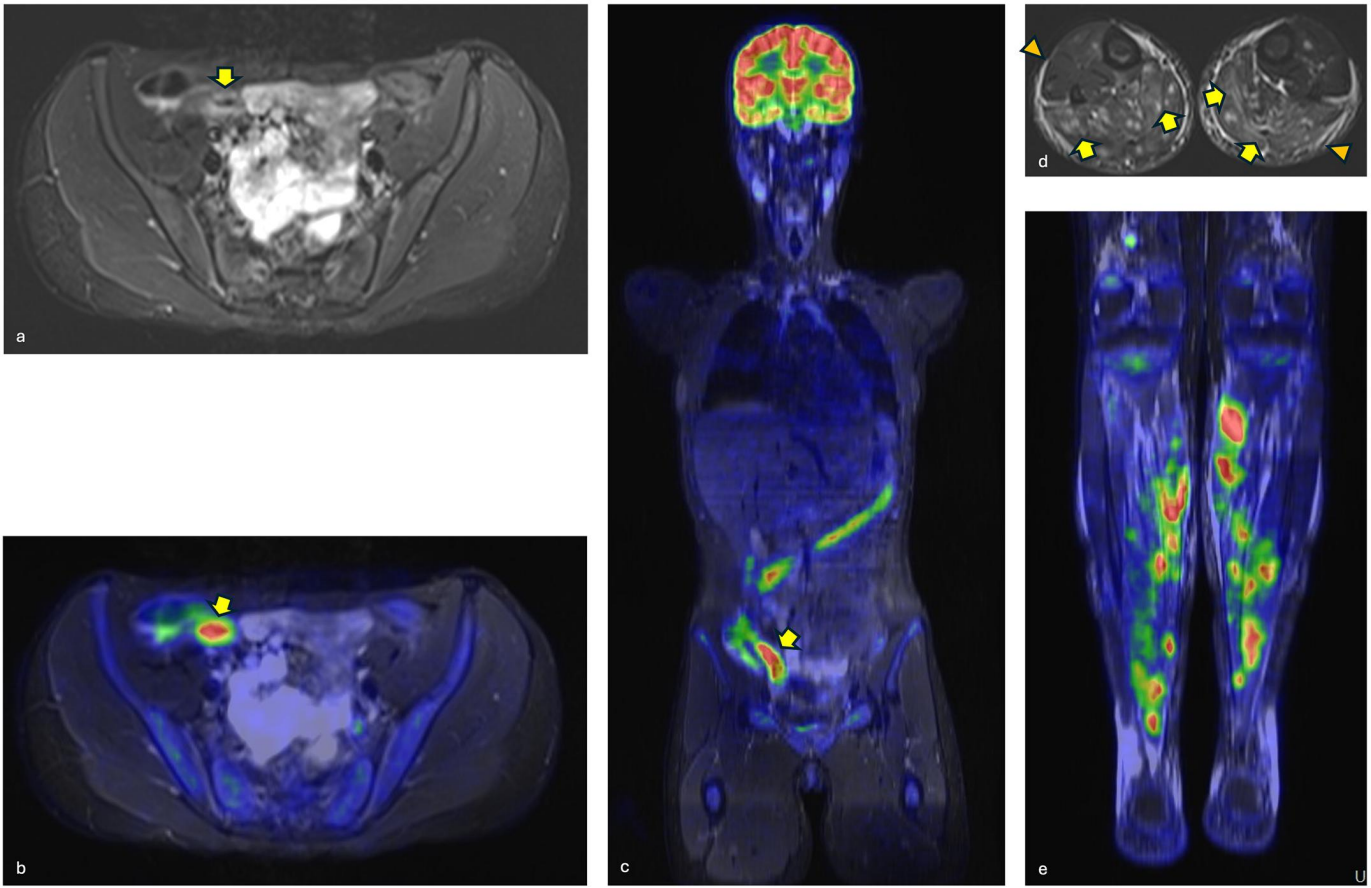
Whole-body [18F]-FDG-PET/MR showing **(a)** the thickened terminal ileal loop (yellow arrow), **(b)** the increased FGD uptake on the fused STIR and PET imaging (yellow arrow) and **(c)** on the coronal view. Diffuse tracer uptake, corresponding to the muscle and fascial edema of the calves (yellow arrow and orange arrowheads, respectively), is evident in **(d)** the axial STIR and **(e)** coronal fused STIR/PET imaging.

The patient remained clinically well for sixteen more months, after which a third similar episode occurred. She again presented with fever (max: 39 °C), sore throat and intense calf pain with walking impairment. The pain intensified during the febrile peaks causing nocturnal awakenings. The laboratory exams confirmed an inflammatory state (Figure 1) as in the previous episodes. A whole-body PET-MRI revealed significant hypermetabolism (SUV max: 5–7) corresponding to areas of edema in the calves, especially in the posterior compartment, associated with peri-fascial edema (Figure 3). Similar but less intense findings were observed in the plantar foot muscles and anterior compartment of the thighs. The terminal ileum was thickened and showed intense FDG uptake (SUV max: 12); mild tracer uptake was evident also in the ascending and transverse colon (Figure 3). Given these findings, further investigations were performed to evaluate the possibility of CD despite the absence of gastrointestinal symptoms. An abdominal ultrasound confirmed the thickening of the terminal ileum and fecal calprotectin levels were significantly elevated at 700 ug/g (normal values <50 ug/g). Conversely, antineutrophil cytoplasmic antibodies (c-ANCA), anti-Saccharomyces cerevisiae antibodies (ASCA), antinuclear antibody (ANA), anti-double strand DNA and extractable nuclear antigen (ENA) antibodies were all negative, as well as the myositis-specific autoantibody panel and HLA-B27 test. A high Sars-CoV2 IgG level (115,000 kBAU/L) indicated a very recent infection. To complete the diagnostic work-up, an endoscopy of the upper and lower digestive tract was performed. Macroscopic examination revealed a hypertrophic, nodular mucosa with hyperplastic polyps of the terminal ileum. Histology showed microerosions, atrophy, and distortion of the villous and cryptal architecture with lymphomonocytic and granulocytic infiltration of the lamina propria. The overall findings were consistent with Crohn's ileitis. A concomitant muscle biopsy detected full-thickness damage of the connective tissue and muscle fibres, with multiple foci of lymphohistiocytes, rare granulocytic infiltrates and degenerative necrotizing changes of myocytes consistent with focal myositis.

Based on the overall clinical picture, a diagnosis of recurrent Gastrocnemius Myalgia Syndrome in the context of otherwise asymptomatic Crohn's Ileitis was established. Therapy with prednisone at 1 mg/kg/day was started, leading to resolution of calf pain and full recovery of ambulation in 48 h. Intestinal inflammation was effectively controlled following induction therapy with corticosteroids, progressively tapered and discontinued in two months, and maintenance treatment with azathioprine. At present, nine months since the treatment started, the patient has not experienced further episodes of myalgia or other symptoms and is in good general condition.

## Discussion

Pediatric-onset IBD typically manifests with gastrointestinal symptoms; however, approximately 10% of children initially present with EIM (7–9). These occur more frequently in CD, affecting up to 30% of patients, compared to around 20% in ulcerative colitis and the most common are arthralgia (17%), aphthous stomatitis (10%) and arthritis (4.4%) (9). Currently, myositis is not listed among the recognized EIMs of IBD. Nonetheless, upon reviewing the literature available on several databases (Pubmed, Embase and Web of Science, search terms included myalgia, myositis, inflammatory bowel disease and Crohn disease, no age or time restrictions) muscular involvement has been reported in adult patients with CD, although it remains a rare and atypical presenting feature (10).

The first case was described by Menard et al. (11) in 1976 in an adult patient. Since then, only 16 additional cases have been reported in the literature, only three of which occurred in pediatric age.

The term Gastrocnemius Myalgia Syndrome (GMS) was first introduced by Christopoulos et al. (5) in 2003 and included myalgia confined to the gastrocnemius muscles as localized form of myositis, serum CPK levels within the normal range and a rapid response to corticosteroid therapy (10).

The normal CPK levels may be explained by the fact that the inflammatory infiltrate predominantly involves the muscle fascia of the gastrocnemius, while sparing the myocytes (10, 12). As concerns the histopathological analysis, the muscle biopsy in GMS shows heterogeneous patterns such as necrotizing focal myositis, as in our patient, granulomatous myositis or rarely vasculitis but only in adult patients (10).

In most reported cases, GMS developed after the diagnosis of CD therefore, in the majority of such cases, intestinal disease was already active at the time of GMS diagnosis (13).

The present case is unique in that GMS preceded the onset of CD by several years, enabling a pre-clinical diagnosis that may have prevented the full clinical expression of IBD. To date, only three pediatric patients with this clinical association have been reported in the literature but in none of them myositis preceded the onset of CD (Table 1).

**Table 1:** Summary of the pediatric cases of gastrocnemius myalgia syndrome and Crohn disease reported to date.

Author, year (Ref.)	Sex	Age at onset (years)	Symptoms at onset	Time interval CD/GMS onset	Clinical features at myositis onset							
					General			Gastrointestinal		Muscular		
					Fever	Sore throat	Weight loss	Abdominal pain	Diarrhoea	Bilateral calf pain	Tenderness	Disability
Co et al. (14)	F	15	Gastro intestinal	8 months earlier	+	+	−	+	+	+	+/−	+
Mogul et al. (15)	M	15	Gastro intestinal	60 months earlier	−	−	−	−	−	+	+	+
Fujita et al. (16)	M	13	Gastro intestinal Myalgias	Concomitant	N/A	N/A	+	+	+	+	−	N/A
Present case	F	15	Myalgias	36 months later	+	+	−	−	−	+	+	+

The first was a 15-year-old female who developed GMS eight months after CD diagnosis (14). She experienced bilateral gastrocnemius pain and difficulty walking, without calf tightness along, in the context of active intestinal disease, with episodes of bloody/mucous diarrhoea and abdominal pain.

The second patient was a 15-year-old boy with a five-year history of CD, who experienced GMS while in clinical remission from CD (15). The most recent reported case is a 13-year-old male patient in whom gastrointestinal and muscular symptoms occurred simultaneously (16). He presented with bloody/mucous diarrhoea, abdominal pain and bilateral calf pain, without impaired gait nor muscle tightness.

In all reported patients, gastrointestinal symptoms appeared either before or concurrently with the onset of muscle symptoms. Conversely, in our case, GMS preceded by years the accidental discovery of CD, in absence of intestinal symptoms. A distinctive feature of our patient's case was the recurrence of GMS episodes, each preceded by upper respiratory tract infections. Indeed, in all three episodes, there was evidence of a recent COVID-19 disease.

In this regard, recent literature indicates that frameshift mutations in the NOD2 gene, such as those associated with CD as in our patient, impair the innate immune response to viral infections by disrupting NOD2's regulatory and signaling functions. NOD2, in fact, is not only a sensor for bacterial peptidoglycan but also participates in antiviral defense by modulating type I interferon responses and interacting with other pattern recognition receptors, including Toll-like receptors (TLRs) (17, 18). Loss-of-function NOD2 mutations result in defective negative regulation of TLR-mediated proinflammatory and type I interferon responses, which can lead to dysregulated inflammation during viral infections (17).

Moreover, SARS-CoV-2 virus is recognized as a potent stimulator of autoimmunity (19) and might have acted as a trigger of the disease onset in a susceptible individual. A recent study in adults demonstrated a clear association between SARS-CoV-2 infection and the subsequent development of autoimmune diseases, including rheumatoid arthritis [adjusted hazard ratio (aHR) of 2.98] and IBD (aHR: 1.78) (9). Angiotensin-converting enzyme 2 (ACE2) receptors are highly expressed on the luminal surface of enterocytes in the small intestine and colon, where they serve as the entry point for SARS-CoV-2 and play a critical role in maintaining gut barrier integrity and regulating the gut microbiome (20–23). In CD, intestinal inflammation is associated with altered ACE2 expression: ACE2 levels are reduced in the small bowel and elevated in the colon, with restoration following anti-cytokine therapy (23–25). This compartmentalized dysregulation may contribute to impaired amino acid transport, increased intestinal permeability, and dysbiosis, all of which are central to Crohn disease pathogenesis (7).

Possible differential diagnoses of conditions potentially involving both the gastrointestinal tract and the muscular system include benign acute childhood myositis (BACM) and polyarteritis nodosa.

BACM is a self-limiting disease typically occurring in children, often triggered by viral infections (most frequently influenza A and B). It usually presents with bilateral calf pain, weakness and refusal to walk and, in most cases, is preceded by prodromal symptoms such as fever (75.3%) and sore throat (26.1%) (26). Unlike BACM, distinct characteristics of GMS are the normal CPK serum level in most patients, gastrointestinal involvement and, particularly in our patient, the recurrence of myalgia episodes.

Polyarteritis nodosa, a systemic necrotizing vasculitis, typically affects medium-sized muscular arteries. Gastrointestinal involvement is reported in 14%–65% of affected patients (27), with a predilection for the small intestine and gallbladder. The most frequent symptom is postprandial ischemic pain, although constipation may also occur. Furthermore, polyarteritis nodosa is often associated with myalgia, sometimes involving calf muscles, but the histological picture is quite different, showing the characteristic middle-sized arteries necrotizing vasculitis (27).

In all reported cases of GMS, corticosteroid therapy has proven effective in rapid muscle pain relief and ambulation recovery. However, relapses during corticosteroid tapering have been described, requiring immunomodulatory drugs to achieve sustained remission (10, 15). With specific reference to our clinical case, the patient is persistently asymptomatic for almost one year since the disease onset. The timely introduction of the immunomodulator during corticosteroid tapering resulted in complete clinical remission. However, a long-term follow-up of the patient will be of great importance in order to monitor for potential relapses of both gastrointestinal and muscular symptoms and to further investigate possible associations.

An intriguing open issue concerns the selective involvement of intestine and calf muscles. Although no clear-cut explanations are evident, possible similarities of the immunohistological composition of gastrocnemius fascia and intestinal tissue are plausible.

In this context, the presence of heterozygous variants in the *NOD2* and *NLRP12* genes, documented in our patient is also noteworthy, as both have been linked to autoinflammatory syndromes with myalgia and intestinal inflammation (28, 29).

In conclusion, our case clearly shows that GMS should be considered among the EIMs of pediatric CD and can precede the onset of intestinal manifestations by years. Therefore, CD should be ruled out in all pediatric patients presenting with predominant, recurrent calf myositis.

## Methods

The Pubmed, Embase and Web of Science databases were searched with no age or time restrictions. General search terms included myalgia, myositis, calf myositis, Crohn disease, Sars-CoV2, extraintestinal manifestation of Crohn's Disease, NOD2, BACM, polyarteritis nodosa, Gastrocnemious Myalgia Syndrome.

## Data Availability

The original contributions presented in the study are included in the article/Supplementary Material, further inquiries can be directed to the corresponding author.
